# Supplementary material for: Content-rich biological network constructed by mining PubMed abstracts
Source: BMC Bioinformatics. 2004 Oct 8;5:147. doi: 10.1186/1471-2105-5-147 (PMC528731; doi:10.1186/1471-2105-5-147)
Supplement: Additional File 2 — The original results of the above study (non-essential files are deleted to keep the file size under the limit set by BMC bioinformatics). [file 1471-2105-5-147-S2.bz2 › chilibotAdditionalFile2/dip05/671ID11110785E11073/html/CCR3_CXCL10.html]

 


 **CCR3** and **CXCL10** 
  
Found 37 abstracts in PubMed, retrieved 05.  
 

 What does Google say? 
 PDF only 
| .edu only 

---

**Interactive relationship** (e.g. stimulation, inhibition, etc)

**Neutral relationship**- **CCR3**  functional responses are regulated by both CXCR3 and its ligands CXCL9,  **CXCL10**  and CXCL11.  Ref: 12884299 Eur J Immunol, 2003

**Non-interactive relationship** (e.g. studied together, co-existance, homology, etc.)

- Consistent with these results, infected cells from WT mice preferentially migrated in response to IP 10  [ **CXCL10** ]  CXCR3 ligand, while those from GKO mice migrated in response to eotaxin CCL11  **CCR3**  ligand.  Ref: 12875978 Am J Pathol, 2003
